# First Seroprevalence Study of Feline Leukemia and Feline Immunodeficiency Infections Among Cats in Algiers (Algeria) and Associated Risk Factors

**DOI:** 10.3390/vetsci11110546

**Published:** 2024-11-06

**Authors:** Fatima Yahiaoui, Moustafa Kardjadj, Meriem Hind Ben-Mahdi

**Affiliations:** 1 Laboratoire de Recherche “Santé et Productions Animales”, Ecole Nationale Supérieure Vétérinaire (ENSV), Oued Smar, Algiers 16200, Algeria; 2 Ecole Nationale Supérieure Vétérinaire (ENSV) d’Alger, Oued Smar, Algiers 16200, Algeria

**Keywords:** FIV, FeLV, seroprevalence, risk factors, Algeria

## Abstract

Feline leukemia and feline immunodeficiency virus infections are two widespread diseases that affect the health of domestic cats all over the world. In Algeria, despite the existence of a large population of cats, there are no published data regarding the epidemiological status of feline retroviruses. Therefore, the objective of the present study was to estimate the seroprevalence of FIV and FeLV infections in cats in Algiers and to assess the main factors associated with the infection. A cross-sectional study was conducted in which 71 cats were sampled from several private veterinary clinics, and their obtained sera were tested using an immunochromatography analysis (SNAP Duo FIV/FeLV Test; Virbac Laboratories, France). The seroprevalence was about 32.39% and 22.53% for FIV and FeLV, respectively. Overall, 11.26% were positive for both infections. The present study showed that FIV seropositivity was associated with a younger age and health status (*p* < 0.05), while FeLV seropositivity was associated with the sex, reproductive status, housing, age, and health status of the cats. This preliminary study, which utilized rapid tests, provides important initial data revealing a relatively high prevalence of retroviruses in cats in Algeria. The results underscore the urgent need for implementing both preventive and management measures to control the spread of these retrovirus diseases. However, to enhance the reliability of future findings, it is essential to expand the sample size and incorporate reference diagnostic methods.

## Simple Summary

Feline leukemia and feline immunodeficiency virus infections are two widespread diseases that affect the health of domestic cats all over the world. This study presents the first seroprevalence investigation of feline leukemia virus (FeLV) and feline immunodeficiency virus (FIV) infections among cats in Algiers, Algeria, filling a significant gap in the regional veterinary literature. A cross-sectional study including 71 cats was conducted, using an immunochromatography analysis (SNAP Duo FIV/FeLV Test; Virbac Laboratories, France). This preliminary study provides important initial data revealing a relatively high prevalence of retroviruses in cats in Algeria. The results underscore the urgent need for implementing both preventive and management measures to control the spread of these retrovirus diseases. However, to enhance the reliability of future findings, it is essential to expand the sample size and incorporate reference diagnostic methods.

## 1. Introduction

Feline leukemia and feline immunodeficiency virus infections are two widespread diseases that affect the health of domestic cats all over the world. The virus of feline immunodeficiency (FIV) and feline leukemia virus (FeLV) belong to the retroviruses family; they are classified, respectively, as lentivirus and γ-retrovirus [1].

Infection with FIV results in the integration of a DNA copy of the viral RNA (called provirus) into the cat’s genome, resulting in lifelong immunodeficiency syndrome, which increases the risk of opportunistic infections in naturally infected cats [2]. FIV, present in saliva and blood, is mainly spread through bites or fight wounds. Other reported modes of transmission include vertical transmission in utero, mucosal infections through sexual contact, and neonatal infections through suckling [2].

Three phases of infection are described: during the first, the cat is viremic and may display some symptoms, mainly peripheral lymphadenopathy. The second phase is the longest, during which the cat is asymptomatic, viral replication is very limited and the animal is clinically healthy. The third and final phase is characterized by an increase in viral replication making the clinical disease more apparent, which is in part due to a CD4+ lymphocytopenia [3].

The feline leukemia virus (FeLV) can lead to the development of tumors, particularly lymphoma, as well as bone marrow suppression syndromes such as anemia and cytopenia [4]. It may also increase susceptibility to secondary infectious diseases due to its suppressive effects on the bone marrow and the immune system [5].

Transmission between cats occurs horizontally, mainly through friendly contacts, during mutual grooming, food sharing and through biting. Blood, feces and breast milk can also be infected with the virus [4,5].

Unlike FIV infection, the different outcome of FeLV infection depends on the interplay of the viral dose and strain and the cat’s immunological response [6]. Some cats infected with FeLV are able to completely clear the infection, they develop an appropriate immune response and eliminate the virus before it progresses beyond local replication in the oropharyngeal tissue (abortive infections) [7], or partially clear the infection (regressive infections); these regressive infections are characterized by transient viraemia, which is followed by the virus becoming latent due to effective humoral and cell-mediated immune responses. Despite the absence of detectable virus in the blood, FeLV proviral DNA may persist in the cat’s cells, which means these cats can act as a source of infection if the virus reactivates [1].

Others become persistently viremic (progressive infections), develop FeLV-associated diseases, and most of them die within a few years after infection [1]. Progressive infection could be linked to mutations in specific viral genes that enhance the efficiency of receptor binding, leading to faster disease onset and severe clinical outcomes [8,9].

Several studies on FIV and FeLV have been conducted all over the world and reported a variable range of prevalence depending on the geographical localization [10]. It ranges (2.34–41%) and (7.7–33.91%) for FeLV and FIV, respectively. In Africa, where the highest rates were reported, only three studies were found in this context: a FelV seroprevalence of 41% was reported in Zimbabwe [11], 0% for FIV and FelV in Ethiopia [12], and 33.91% for FIV and 8% for FeLV in Egypt [13].

In Algeria, despite the existence of a large population of pets and stray cats, no protective vaccination program has been implemented except for rabies. To our knowledge, there are no published data regarding the epidemiological status of feline retroviruses in Algeria. Therefore, the objective of the present study was to estimate the seroprevalence of FIV and FeLV infections in cats in Algiers and to assess the main factors associated with the infection.

## 2. Materials and Methods

Population samples and epidemiological data

A cross-sectional study was conducted in the central and eastern parts of Algiers between March 2019 and February 2020. A total of 71 cats were sampled from various private veterinary clinics, which were randomly selected to ensure that the owned cat population accurately reflects the broader cat population in Algiers.

Cat owners were informed about the study’s objectives, and their consent was obtained prior to participation.

The study was approved by the scientific and ethical council of laboratory research of the National High Veterinary School of Algiers. All veterinarians adhered to best practices in line with WOAH (formerly OIE) ethical guidelines and animal welfare regulations, ensuring the humane treatment of the animals throughout the study. Blood samples were obtained by jugular venipuncture, and sera were obtained by centrifugation at 3000 rpm for 5 min and then stored at −20 °C until analysis.

Data on sex, reproductive status, outdoor access, housing conditions (categorized as street, shelter, or household cat), breed, age, and health status were collected for the risk factor analysis of FIV and FeLV infections. Housing conditions were defined as follows: street cats were free roaming without a permanent home, shelter cats resided in animal shelters, and household cats were owned and lived primarily indoors. Outdoor access was defined as whether a cat had unrestricted or occasional access to the outdoors regardless of its primary housing condition. Cats were classified as “sick” if they exhibited clinical signs such as weight loss, lethargy, loss of appetite, or generalized lymphadenopathy, while those with no observable clinical signs were classified as “healthy”.

Serological testing

The obtained sera were tested using an immunochromatography analysis (SNAP Duo FIV/FeLV Test; Virbac Laboratories, Carros, France). This assay detects antibodies of the transmembrane glycoprotein (gp40) to the FIV and FeLV core viral capsid protein antigen (p27) (the diagnostic sensitivity and specificity of the test were 97.3% and 98.6% for FIV respectively, and 94.7% and 99.2% for FeLV, respectively).

Statistical analysis

For the risk factor analysis, we initially conducted a univariable exploratory analysis to select variables with a *p*-value ≤ 0.2 using either the chi-square test or Fisher’s exact test. Prior to proceeding with multivariable logistic regression, we addressed potential collinearity by performing a correlation analysis among the independent variables. Variables with a correlation coefficient > 0.9 were excluded based on biological plausibility to ensure a robust model. The remaining variables were then subjected to multivariable logistic regression. The fit of the final model was assessed using the Hosmer–Lemeshow test. Confounding effects were evaluated by observing changes in the regression coefficients when new variables were added; changes exceeding 20% were considered indicative of confounding. All calculations were performed using R (4.1.0) and RStudio (1.4.1717) for Windows. All statistical tests were two-sided with significance set at *p* < 0.05 [14,15].

## 3. Results

Out of the 71 cats tested, 23 (32.39%) were positive to FIV antibody test, and 16 (22.53%) were positive to FeLV antigen test (Table 1).

FIV Infection

The study showed no significant difference in FIV seroprevalence based on sex and reproductive status (*p* > 0.05). In our analysis, we found that the prevalence of FIV/FeLV among both indoor cats and purebred cats was 0%. As a result, no statistical tests could be performed to evaluate differences related to outdoor access and breed, since the absence of positive cases in these groups limits the ability to make meaningful statistical comparisons.

The FIV prevalence by sex was 33.33% (13/39) for males and 31.25% (10/32) for females (*p* = 0.654), and by reproductive status, it was 33.33% (12/36) in neutered cats and 31.42% (11/35) in intact cats (*p* = 0.619) (Table 1).

Age-wise, FIV was most prevalent in cats aged 4 to 6 years (43.47%, 10/23), which was followed by those younger than 1 year (31.81%, 7/22) and those aged 2 to 3 years (26.31%, 5/19) (*p* = 0.029).

FeLV Infection

Concerning FeLV infection, the prevalence was higher in males (25.64%, 10/39) compared to females (18.75%, 8/39) (*p* = 0.035). Additionally, FeLV prevalence was higher in intact cats (25.71%, 9/35) than in neutered ones (19.44%, 7/36) (*p* = 0.041).

Regarding housing, the prevalence of both diseases was higher in shelter cats. FIV prevalence was 42.10% (8/19) in street cats, 47.36% (9/19) in shelter cats, and 18.18% (6/33) in house cats (*p* = 0.034). FeLV prevalence was 5.26% (1/19) in street cats, 57.89% (11/19) in shelter cats, and 12.12% (4/33) in house cats (*p* = 0.016).

FeLV-positive cats were mostly aged 2 to 3 years (36.84%, 7/19) and 4 to 6 years (26.08%, 6/19) with 3 positive cats younger than 1 year (*p* = 0.018).

Sick cats showed a higher prevalence of both infections compared to healthy ones (*p* = 0.015 for FIV and *p* = 0.001 for FeLV) with 51.61% (16/23) for FIV and 48.38% (15/16) for FeLV.

FeLV and FIV risk factor analysis

The multivariable regression study demonstrates that the risk of both infections can be increased when associated with different factors (Table 2).

Multivariable regression analysis indicated that the risk of FIV infection was significantly higher in street and shelter cats, being 2.33 and 2.48 times greater, respectively, than in house cats (95% CI: 1.74–2.99, *p* = 0.038; 95% CI: 1.92–3.58, *p* = 0.034). Additionally, sick cats had a 2.84 times higher risk of FIV infection compared to healthy cats (95% CI: 1.66–3.92, *p* = 0.035).

For FeLV, males and intact cats had higher infection risks with males having a 1.51 times higher risk (95% CI: 1.09–2.33, *p* = 0.035) than female and intact cats having a 1.39 times higher risk (95% CI: 1.11–1.88, *p* = 0.048) than neutered cats. Shelter cats were 2.88 times more likely to be FeLV positive than street and house cats (95% CI: 2.21–3.59, *p* = 0.031). FeLV positivity was also higher in cats aged 2 to 3 years (2.95 times higher, 95% CI: 1.88–3.72, *p* = 0.029) and 4 to 6 years (2.14 times higher, 95% CI: 1.35–3.66, *p* = 0.028) than other groups (age <1 and >7 years). Sick cats had a 3.22 times higher risk of FeLV positivity compared to healthy cats (95% CI: 1.51–5.62, *p* = 0.031).

Co-infection prevalence and risk factor analysis

Out of the 71 cats tested, 8 (11.26%) were tested positive for both infections (Table 3).

Co-infection risk was found to be 2.57 times higher in neutered cats (95% CI: 1.45–3.81, *p* = 0.027) and 6.22 times higher in shelter cats (95% CI: 2.66–11.35, *p* = 0.034) (Table 3).

## 4. Discussion

This study represents the first assessment of FIV and FeLV infections in cats in Algeria, revealing a prevalence of approximately 32.39% for FIV and 22.53% for FeLV, with 11.26% of cats testing positive for both infections. These findings highlight the significance of understanding retrovirus prevalence within the context of Algeria, especially given the absence of prior data.

Numerous studies on retroviral diseases have been conducted worldwide, revealing significant variations in prevalence based on geographical regions. In the United States, the prevalence of FeLV, as determined by antigenemia testing, ranges from 2.3% to 3.3%. In Europe, it varies from 0% to 15.6%, while in South America, the rates are between 3.0% and 28.4%. In Asia and Australia/New Zealand, the prevalence ranges from 0% to 24.5% [8,9,10,11,12,13,14,15,16].

Lower prevalence rates are typically found in developed countries, such as the USA, Canada, the United Kingdom, and several European nations, with FeLV rates ranging from 2.3% to 3.6% and FIV rates from 2.5% to 5.2%. These lower figures are likely attributable to effective population control and vaccination programs [17]. In contrast, higher rates have been documented in Italy, with 7.64% for FeLV and 8.32% for FIV [18], and in Spain, where the rates are 4.39% for FeLV and 19.30% for FIV [19]. The elevated prevalence in these countries may be linked to a larger population of outdoor free-living cats [10,11,12,13,14,15,16,17,18,19,20].

Conversely, higher seroprevalences have been reported in Türkiye (25.2% for FIV and 45.2% for FeLV) [21], as well as in various developing countries across Asia and South America, where FIV prevalence ranges from 5.8% to 44%, and FeLV prevalence spans from 4.2% to 28% [10,11,12,13,14,15,16,17,18,19,20,21,22].

The seroprevalence of FeLV in the cat population studied in Algeria was found to be 22.53%, which is significantly higher than the 13.84% and 8% reported in Lebanon and Egypt [13,14,15,16,17,18,19,20,21,22,23,24,25,26]. However, this prevalence is lower than the rates of 44% and 45.2% observed in Zimbabwe and Turkiye, respectively [11,12,13,14,15,16,17,18,19,20,21]. These discrepancies may arise from differences in study design and diagnostic methodologies. Our study encompassed both household and stray cats, while the Turkish study concentrated exclusively on stray cats and specifically evaluated the FeLV antibody rate. The inclusion of diverse populations in our research allows us to analyze the risk factors more comprehensively, including the context of outdoor access for different age groups. The findings suggest that free-roaming cats, regardless of age, may be at a higher risk of infection [21].

The current study examined a diverse population of cats, encompassing various genders, age groups, health statuses, and lifestyles. Our findings revealed that the seroprevalence of FIV was significantly influenced by several factors, particularly the age, housing, and health of the cats. Notably, we observed higher seroprevalence rates in cats aged 2 to 3 years and 4 to 6 years compared to younger cats. This increase in seroprevalence could be attributed to the increased likelihood of exposure to infected cats during outdoor access, where interactions—such as fights—are more common. Furthermore, as FIV is a lifelong infection, it is expected that the seroprevalence would rise in older cohorts over time [23,24].

Additionally, our results indicated that street and shelter cats had significantly higher FIV seroprevalence rates than indoor cats. Outdoor cats are more prone to engage in territorial disputes and mating behaviors, increasing their risk of infection. Cats in shelters often come from outdoor environments, further heightening their exposure to FIV [20].

In contrast, a study conducted by Sprißler et al. [22], which focused solely on healthy cats, reported a much lower prevalence compared to studies that included sick cats [24]. Our findings also highlight that sick cats had a higher seroprevalence of both FIV and FeLV compared to their healthy counterparts, underscoring the impact of retrovirus infections on feline health and the significance of health status in infection detection [25].

Overall, the health status of cats emerges as a critical risk factor for retrovirus infections, especially in endemic environments. This study marks the first documentation of retrovirus infections in Algeria, contributing valuable insights into feline health in the region.

Our analysis indicates that intact cats are at a higher risk of FeLV infection compared to neutered cats. This observation aligns with findings from several studies that demonstrate a link between neutering and a reduced incidence of retrovirus infections in domestic cats [26,27]. The increased seropositivity rate among intact cats can likely be attributed to their territorial aggression and free-roaming behaviors, which elevate their chances of coming into contact with infected cats.

In our study, male cats exhibited a higher risk of FeLV exposure (25.64%) than female cats (18.75%) (*p* = 0.035), suggesting that the habits associated with males, particularly their outdoor behaviors, contribute to this increased vulnerability [28]. Additionally, our results indicate that similar to FIV infections, FeLV prevalence is influenced by factors such as the age, health status, and housing conditions of the cats.

Interestingly, the prevalence of FeLV was found to be higher in shelter cats compared to stray cats. This may be due to the proximity of cats within shelters, which facilitates horizontal transmission of the virus through interactions such as mutual grooming, food sharing, and biting.

Despite these findings, our study has several limitations. First, the results were not confirmed through Western blot assays or PCR tests, which are recommended as confirmatory methods for detecting FIV and FeLV positivity. Only an antigen test was performed for FeLV, which detects cats with progressive infections and sometimes those with early regressive infections, cats with regressive and abortive infections may have been missed [29]; additionally, the FeLV p27 antigen in the serum may fall below the detection limit of the test, raising the potential for false positives and negatives. Future studies should incorporate additional confirmatory tests to enhance accuracy.

Furthermore, it should be noted that the administration of FIV and FeLV vaccines could introduce a potential bias in the testing outcomes [30]. Nonetheless, since vaccines against FIV and FeLV are unavailable in Algeria, no bias related to immunization could be present in our study population.

The sample size of 71 cats is relatively small, which limits the generalizability of our results and the statistical power, particularly for multivariate analyses. The absence of positive cases in specific subgroups (e.g., indoor and purebred cats) precluded statistical testing for some risk factors, such as outdoor access and breed. Moreover, our cross-sectional study design identifies associations but does not establish causality. Although we addressed various risk factors, including housing conditions and health status, more refined categories and larger sample sizes would facilitate a more comprehensive analysis in future research.

## 5. Conclusions

To the best of our knowledge, this study represents the first investigation into the seroprevalence of FIV and FeLV among the cat population in Algeria, revealing a relatively high prevalence of both viruses. These findings enhance our understanding of the occurrence and circulation of FIV and FeLV in the region, highlighting the urgent need for measures to control their spread. Given the significant impact these diseases have on immune system function, it is critical to promote both preventive and management strategies for retrovirus infections. The compromised immunity associated with retroviral infections can facilitate the spread and worsening of other diseases, underscoring the necessity for comprehensive health interventions in the feline population.

## Figures and Tables

**Table 1:** Seroprevalence of FIV and FeLV infection in Algerian cats.

			FIV Infection			FeLV Infection		
Variable	Category	Tested	Positives	Prevalence%	*p* Value	Positives	Prevalence%	*p* Value
Sex	Male	39	13	33.33		10	25.64	
	Female	32	10	31.25	0.654	6	18.75	0.035 *
Reproductive status	Neutered	36	12	33.33		7	19.44	
	Intact	35	11	31.42	0.619	9	25.71	0.041 *
Access to outdoor	Yes	46	23	50		16	34.78	
	No	25	0	0	/	0	0	/
Housing	Street	19	8	42.10		1	5.26	
	Shelter	19	9	47.36		11	57.89	
	House	33	6	18.18	0.034 *	4	12.12	0.016 *
Breed	Purebred	12	0	0		0	0	
	Mixed-breed	59	23	38.98	/	16	27.11	/
Age	0–1 year	22	7	31.81		3	13.63	
	2–3 years	19	5	26.31		7	36.84	
	4–6 years	23	10	43.47	0.029 *	6	26.08	0.018 *
	≥7 years	7	1	14.28		0	0	
Health status	Healthy	40	7	17.5		1	2.5	
	Sick	31	16	51.61	0.015 *	15	48.38	0.001 *

* statistically significant.

**Table 2:** FIV and FeLV risk factors analysis.

			FIV Infection					FeLV Infection				
Variable	Category	Tested	Positives	Prevalence%	OR	95% IC	*p* Value	Positives	Prevalence%	OR	95% IC	*p* Value
Sex	Male	39	13	33.33	/	/	/	10	25.64	1.51	(1.09–2.33)	0.035 *
	Female	32	10	31.25	/	/		6	18.75	Ref		
Reproductive status	Neutered	36	12	33.33	/	/	/	7	19.44	Ref		
	Intact	35	11	31.42	/	/		9	25.71	1.39	(1.11–1.88)	0.048 *
Access to outdoor	Yes	46	23	50	/	/	/	16	34.78	/	/	/
	No	25	0	0	/	/		0	0	/	/	/
Housing	Street	19	8	42.10	2.330	1.74–2.99	0.038 *	1	5.26	0.88	(0.55–1.26)	0.094
	Shelter	19	9	47.36	2.48	1.92–3.58	0.034 *	11	57.89	2.88	(2.21–3.59)	0.031 *
	Housed cat	33	6	18.18	Ref			4	12.12			
Breed	Purebred	12	0	0	/	/		0	0	/	/	
	Mixed-breed	59	23	38.98	/	/		16	27.11	/	/	
Age	0–1 year	22	7	31.81	Ref	/		3	13.63	Ref	/	
	2–3 years	19	5	26.31	1.12	0.41–1.65	0.32	7	36.84	2.95	(1.88–3.72)	0.029 *
	4–6 years	23	10	43.47	1.87	1.22–2.69	0.041 *	6	26.08	2.14	(1.35–3.66)	0.028 *
	≥7 years	7	1	14.28	/			0	0	/	/	/
Health status	Healthy	40	7	17.5	Ref			1	2.5	Ref		
	Sick	31	16	51.61	2.84	1.66–3.92	0.035 *	15	48.38	3.22	(1.51–5.62)	0.031 *

* statistically significant; OR: odds ratio; IC: interval of confidence.

**Table 3:** Co-FeLV-FIV infection risk factor study.

Variable	Category	Tested	Positives	Prevalence%	OR	95% IC	*p* Value
Sex	Male	39	5	12.82	/	/	/
	Female	32	3	9.37	/	/	/
Reproductive status	neutered	36	6	16.66	2.57	(1.45–3.81)	0.027 *
	Intact	35	2	5.71	Ref		
Access to outdoor	yes	46	8	17.39	/	/	/
	no	25	0	0	/	/	/
Housing	street	19	0	0	/		
	shelter	19	7	36.84	6.22	2.66–11.35	0.034 *
	House cat	33	1	3.03	Ref		
Breed	Purebred	12	1	8.33	/	/	/
	Mixed-breed	59	7	11.86	/	/	/
Age	0–1 year	22	1	4.54	Ref		
	2–3 years	19	3	15.78	3.11	1.54–4.88	0.041 *
	4–6 years	23	4	17.39	3.81	2.14–6.38	0.035 *
	≥7 years	7	0	0	/	/	/
Health status	Healthy	40	0	0	/	/	/
	Sick	31	8	25.80	/	/	/

* statistically significant; OR: odds ratio; IC: interval of confidence.

## Data Availability

The datasets collected/generated during and/or analyzed during the current study are available on reasonable request to the corresponding author.
